# Identification of the Best Predictive Model for Mortality in Outborn Neonates—Retrospective Cohort Study

**DOI:** 10.3390/healthcare11243131

**Published:** 2023-12-09

**Authors:** Maria Livia Ognean, Bianca Coțovanu, Dumitru Alin Teacoe, Ioana Andrada Radu, Samuel Bogdan Todor, Cristian Ichim, Iris Codruța Mureșan, Adrian-Gheorghe Boicean, Radu Galiș, Manuela Cucerea

**Affiliations:** 1 Faculty of Medicine, Lucian Blaga University Sibiu, 550169 Sibiu, Romania; maria.ognean@ulbsibiu.ro (M.L.O.); samuelbogdant@gmail.com (S.B.T.); cristian.ichim@ulbsibiu.ro (C.I.); iris.muresan@ulbsibiu.ro (I.C.M.); adrian.boicean@ulbsibiu.ro (A.-G.B.); 2 Department of Neonatology, Clinical County Emergency Hospital Sibiu, 550245 Sibiu, Romania; cotovanubianca@gmail.com; 3 Department of Neonatology, Clinical County Emergency Hospital Bihor, 410167 Oradea, Romania; radu.galis@scjubh.ro; 4 Department of Neonatology, George Emil Palade University of Medicine, Pharmacy, Science, and Technology, 540142 Targu Mures, Romania; manuelacucerea@yahoo.com

**Keywords:** newborn, outborn, scoring system, sick neonatal score, mortality, neonatal transportation

## Abstract

Background: Transportation of sick newborns is a major predictor of outcome. Prompt identification of the sickest newborns allows adequate intervention and outcome optimization. An optimal scoring system has not yet been identified. Aim: To identify a rapid, accurate, and easy-to-perform score predictive for neonatal mortality in outborn neonates. Material and methods: All neonates admitted by transfer in a level III regional neonatal unit between 1 January 2015 and 31 December 2021 were included. Infants with congenital critical abnormalities were excluded (N = 15). Gestational age (GA), birth weight (BW), Apgar score, place of birth, time between delivery and admission (AT), early onset sepsis, and sick neonatal score (SNS) were collected from medical records and tested for their association with mortality, including in subgroups (preterm vs. term infants); GA, BW, and AT were used to develop MSNS-AT score, to improve mortality prediction. The main outcome was all-cause mortality prediction. Univariable and multivariable analysis, including Cox regression, were performed, and odds ratio and hazard ratios were calculated were appropriate. Results: 418 infants were included; 217/403 infants were born prematurely (53.8%), and 20 died (4.96%). Compared with the survivors, the non-survivors had lower GA, BW, and SNS scores (*p* < 0.05); only the SNS scores remained lower in the subgroup analysis. Time to admission was associated with an increased mortality rate in the whole group and preterm infants (*p* < 0.05). In multiple Cox regression models, a cut-off value of MSNS-AT score ≤ 10 was more precise in predicting mortality as compared with SNS (AUC 0.735 vs. 0.775) in the entire group and in the preterm infants group (AUC 0.885 vs. 0.810). Conclusions: The new MSNS-AT score significantly improved mortality prediction at admission in the whole study group and in preterm infants as compared with the SNS score, suggesting that, besides GA and BW, AT may be decisive for the outcome of outborn preterm infants.

## 1. Introduction

One of the targets of the World Health Organization (WHO)’s Millennium Development Goal (MDG) is the reduction of the under-five years of age mortality by two-thirds [1]. Neonatal mortality represents a significant part of under-five mortality. Reducing the neonatal mortality rate is a key factor for reaching the MGD goal, as a recent WHO report underlines that the neonatal mortality rate, both globally and in Romania, did not show a similar significant decline as global child mortality from 2015 to 2019 [2].

The neonatal mortality rate is an important indicator of the health and economic status of a nation [3]. Most neonatal deaths occur within the first 7 days of life. Delayed neonatal transportation and inefficient care during transfer are significant risk factors for neonatal mortality [4,5,6]. Neonatal transportation of sick newborns is a major predictor of outcome. This fact is often neglected in low- and middle-income countries [6,7], as it is in Romania. In-utero transportation of high-risk pregnancies, according to regionalization of maternal and neonatal care, is the safest option for both maternal and neonatal outcomes [8,9,10,11]. Unfortunately, preterm delivery and delivery of sick neonates are not always easy to anticipate and will continue to occur at lower-level institutions and even at home. Efforts to improve neonatal stabilization pre-transport, neonatal care during transport and the neonatal transport system itself—organization, training, equipment—are challenging in many countries. Outborn neonates are facing significantly higher morbidity and mortality rates [8,11,12].

Early recognition of sick neonates, optimal resuscitation if needed, prompt recognition, and immediate competent interventions are needed to treat hypoglycemia, seizures, and respiratory distress, prevention and treatment of hypothermia, hypoxia, hypotension, adequate monitoring before and during neonatal transport, and rapid transfer are extremely important for optimization of neonatal outcome [5,8,9,13,14,15,16]. A regionalized, specialized neonatal transport system may reduce neonatal complications and improve survival rates [11,14,17,18]. For many years now, clinicians have tried to evaluate the outborn infants at admission to identify and promptly address all complications that may impact their prognosis, as the severity of the disease was described as an important prediction factor for mortality in newborn infants [19,20,21]. Severity scores were developed accordingly, including clinical and laboratory parameters, with different utilities and results in predicting mortality and morbidity rates [22]. Additionally, not all these scores can be applied in resource-limited areas. However, an optimal score has not yet been identified, and it may still be challenging to do so. This is because the validation of these scores has not shown the same level of sensitivity and specificity across various neonatal populations, regions, countries, and units. Additionally, there are many organizational differences between national maternal and neonatal care, regionalization, and neonatal transport systems [5,23,24,25]. An ideal score—or predictive model for the severity of the disease–should be easy, applicable early after admission, needing a minimum of invasive procedures, reproducible, with a good ability to predict mortality and specific morbidities and to discriminate between neonates with different outcomes [9,21,22,25,26,27,28]. Additionally, these predictions allow better planning and usage of resources of care, improvements of neonatal care before and during transport, cost analysis, evaluation of the care quality, comparisons between neonatal units, research, and parental counseling [22,25,26,27,28,29].

The organization of the Romanian neonatal transport system started in 2004, immediately after the regionalization of maternal and neonatal care in 2002, but still lacks, in many parts of the country, trained specialized staff and special equipment. Considering the improved rates of survival at lower gestational ages and the insufficient number of beds in neonatal intensive care units (NICU), we face a continuous need for better critical neonatal care, as survival is not the ultimate goal of NICU care anymore. Based on a prospective cohort, using the sick neonatal score (SNS) [14,30] and statistical models, our study aimed to identify a rapid and easy-to-perform score predictive for all-cause neonatal mortality in newborns submitted to our unit after delivery. Sick neonatal score (SNS) developed by Rathod et al. [14,30] was used initially to compare the survivors and non-survivors in our study group. New variables—gestational age (GA), birth weight (BW), gender, Apgar score, place of birth, time between delivery and admission (AT), and early onset sepsis—were tested for their relevance in predicting all-cause mortality in preterm and term neonates, as well in the entire study group. Variables associated with mortality were used to create a new predictive score, and its accuracy was verified for the entire study group and in preterm and term infants. All these steps are described below in the paper.

## 2. Materials and Methods

**Figure 1:**
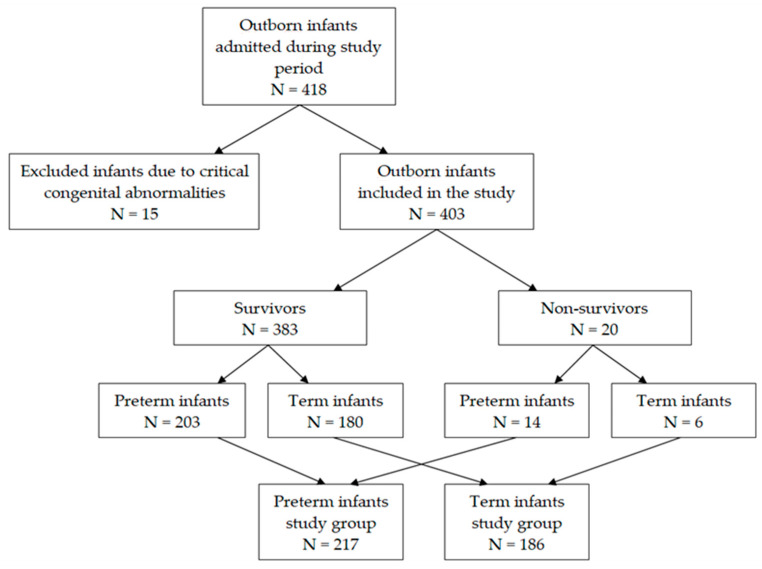
Flow chart for selecting study groups.

The study included all neonates (N = 418), irrespective of gestational age, consecutively admitted by transfer in level III regional neonatal unit of the Clinical County Emergency Hospital Sibiu, Romania, between 1 January 2015 and 31 December 2021, representing 1.95% of all admissions during the study period (N = 21,412). Newborns with critical congenital defects (N = 15) were excluded (Figure 1). Neonatal and NICU charts were used to extract data. Sick neonatal score was calculated for every newborn using the criteria suggested by Rathod et al. [30] (Table 1) but using the first blood glucose (we considered this as more relevant for infants’ condition as intravenous therapies may modify blood glucose levels) and rectal temperature at admission (as this is more accurate and part of our local protocol for evaluation of outborn infants). We also collected data in regards gestational age (defined using either the best obstetrical estimate based on first-trimester ultrasound or the date of the last menstrual cycle), birth weight, gender, the time between birth and arrival to our unit (in hours) (referred below as admission time or time to admission), Apgar score at 1 min, diagnosis of early-onset sepsis, and place of birth (defined home delivery, delivery at level I, II, or III neonatal units). Per protocol unit, all these data must be completed by nurses and physicians in neonatal records. According to current regulations in Romania, level I neonatal units must transfer all infants with a birth weight less than 2500 g, even healthy ones and all sick infants, to higher levels. Level II units have the competency, experience, and equipment necessary to care for infants with a birth weight greater than 1500 g and/or a gestational age greater than 32 weeks, as long as they do not require mechanical ventilation or other invasive procedures. All other newborns must be transferred to level III units (the highest level of neonatal care) [31,32].

Outborn infants (403 included in the final analysis) were classified into two groups—survivors and non-survivors—according to the primary outcome, all-cause mortality. Subgroup analyses were performed, as the study group was also classified according to gestational age in preterm (N = 217) and term infants (N = 186), respectively. All the collected variables, including SNS score, were compared between infants who survived at discharge from the maternity hospital and those who died.

We had chosen the sick neonate score (SNS) score, a score initially developed by Hermansen in 1994 [33] and modified by Rathod et al. [30] because it was validated both in high-income and in resource-limited countries [30,33] and did not require invasive procedures, can be performed rapidly, using easy to measure variables and widely available tools. The accuracy of the SNS score was also extensively tested with good or satisfactory results in the literature. The variables used for SNS calculation are presented in Table 1, but random glucose level was replaced in our study with the first glucose level measured after admission, and axillary temperature was replaced by rectal one. Prematurity was defined as birth occurring before completing 37 weeks of gestation. Blood glucose levels were measured using a blood gas analyzer. The first reading was used to calculate the SNS. An electronic thermometer was used to measure rectal temperature at admission. Respiratory effort and capillary refill time evaluated, scored, and noted by physicians at admission were used for SNS calculation; heart frequency and peripheral oxygen saturation were read and registered on a pulse oximeter (using Masimo^®^ technology) also at admission, in room air. Peripheral oxygen saturation is a rapid, non-invasive indicator of hypoxia; therefore, it is measured at admission and continuously monitored in all the newborns cared for in the neonatal intensive care unit starting the minute the infants are admitted. Non-invasive mean blood pressure measurement was measured upon admission, and the first values were used for SNS calculation. Early onset sepsis was defined as an increased level of inflammatory markers (C-reactive protein and/or procalcitonin) associated with clinical signs and symptoms of infection occurring in the first 72 h of life.

Because most of the continuous variables did not have a normal distribution, they are presented both as median (and standard deviation, SD) and as median (interquartile range, IQR) but were compared with the Mann–Whitney U test. Spearman’s coefficient of correlation was used to highlight relationships between variables. Categorical variables were presented as counts (percentages) and compared to the Pearson Chi-square test. The receiver’s operating characteristics (ROC) curves were used to calculate the area under the curve (AUC) for each independent continuous variable and to establish optimal cut-off points. They are presented along with their sensitivity and specificity values. Survival analysis was conducted using Kaplan–Meyer curves, which consisted of presenting the survival curves and rank log-rank test, and through univariable and multivariable Cox proportional hazards model (Cox regression). To evaluate the goodness-of-fit for each model and to establish hierarchies, we saved the hazard function for each Cox regression model performed and tested if the hazard probabilities matched the outcome by running the ROC curves. The AUC of each model was indicative of its accuracy in outcome prediction. Two-sided Confidence intervals of 95% were presented for AUC and hazard ratios (HR). A *p*-value < 0.05 was considered statistically significant. Statistical analysis was performed using IBM SPSS version 22.0.

## 3. Results

### 3.1. Group and Subgroup Analysis

**Figure 2:**
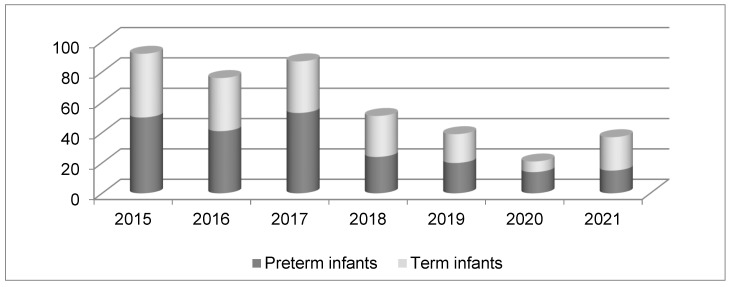
Representation of the number of transferred newborn infants during the study period.

The study group consisted of 418 newborns submitted to our unit during the study period: 15 of them were excluded from the analysis because they had been transferred due to severe congenital abnormalities; 224 (55.6%) were males; and 20 neonates died before discharge (4.96%). The number of transfers decreased significantly after 2017 and is presented in Figure 2. The baseline characteristics of the study group and subgroups based on gestational age are presented in Table 2.

At first, the SNS in our cohort was verified not only for the entire cohort but also in subgroups based on gestational age, and the results for the mean values (SD) are presented in Table 2. The collected variables identified in the literature as important factors predictive of mortality rate: gestational age, birth weight, the time duration between birth and admission to our unit, birth asphyxia (as reflected by an Apgar score at 1 min), and early onset sepsis diagnosis were analyzed for the entire study group and for the subgroups based on gestational age (preterm vs. term infants), for survivors and non-survivors and are presented in Table 3 and Table 4. As shown in Table 3, mean values (SD) for gestational age, birth weight, Apgar score at 1 min, and SNS score are lower in the non-survivors preterm infants’ group and in the whole study group as compared with survivors, while time to admission was increased in deceased infants in all the groups as compared with survivors. Mann–Whitney T was used to verify associations between the collected variables as statistical analysis has demonstrated an abnormal distribution of data values (Table 4). All comparisons were performed in search of variables with the most significant impact on mortality in our population. The study found that mortality rates are significantly linked to gestational age and birth weight for the overall group but not for preterm and term infants separately. Time to admission is a significant factor for mortality in both preterm infants and the overall group, while a lower Apgar score at 1 min and a lower SNS score is significantly associated with mortality across all groups (Table 3 and Table 4). No associations are found for gender, Apgar score < 3 at 1 min, and early sepsis (Table 5). Most of the infants who died, especially premature ones, were transferred from level II neonatal units (Table 5).

### 3.2. SNS Score to Predict Mortality

**Figure 3:**
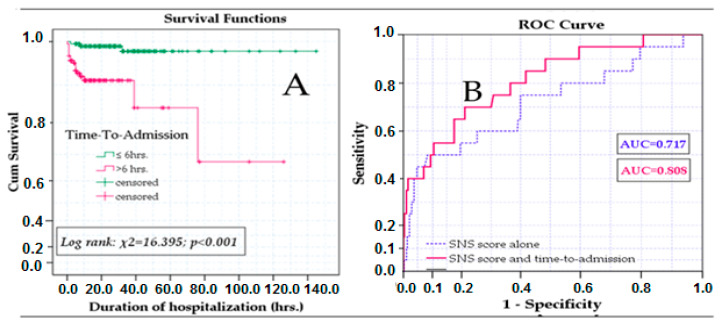
(**A**) Survival differences between newborns based on the time-to-admission period. (**B**) Improvement of hazard estimation by adding a time-to-admission variable to the SNS score regression model.

An ROC curve was performed to establish cut-off points for the time duration between birth and admission, which was found to be significantly associated with mortality. The area under the curve (AUC) is 0.664, *p* = 0.013. The optimal cut-off point for the time to admission variable is 6.5 h (sensitivity 85%, specificity 54%). Consequently, the patients were separated into two groups based on the cut-off point, and a Kaplan–Meyer curve and a log test were conducted; these tests showed significant differences in survival between newborns admitted before and after 6.5 h after birth (log-rank test: that χ^2^ = 16.395; *p* < 0.001) (Figure 3A). Univariable and multivariable Cox regression indicated that time to admission over 6.5 h (hazard ratio (HR): 14.009; CI 95%: 3.794–51.724; *p* < 0.001) and SNS score (HR: 0.664, CI 95%: 0.574–0.769; *p* < 0.001) were predictive for mortality outcome; adjustments for birth weight and gestational age were made in multivariable analysis. When the time to admission was added to the SNS score in multivariable Cox regression, we concluded that death HR became more accurate (AUC 0.808 vs. 0.717) (Figure 3B). These results encouraged us to include time to admission in a modified scoring system, along with gestational age and birth weight, in our attempt to improve the mortality prediction in our population and settings.

### 3.3. Use of an Improved SNS Score for Predicting Mortality

To evaluate the inclusion of time-to-admission on survival rates we tried to modify SNS score to include the three variables with significant impact on mortality in our study group. We classified patients into three different groups based on the time-to-admission value: patients admitted in less than 6 h after birth were classified as early admitted; patients admitted between 6–12 h were considered intermediately admissions, and those admitted after ≥12 h were late admissions. Kaplan–Meyer curve and log-rank test show significant differences in survival between these groups (χ^2^ (2) = 14.679; *p* = 0.001). A scoring system was established for each of these groups based on the results of Cox regression coefficients. Compared with the intermediate group (6–12 h), patients early admitted have a 1.347 higher survival rate, while compared with late admissions (≥13 h) the survival rate is 3.278 higher. Therefore, in the new scoring system, time-to-admission was granted 3 points for early admission, 1 point for intermediate admission, and 0 points for admissions after 13 h. Additionally, we added gestational age and birth weight into the scoring system, using the same evaluation as in the Modified SNS (Table 6). Consecutively, we calculated a Modified SNS-Admission Time score (MSNS-AT score) for all our patients by adding the values for the three new variables to SNS scores. Ultimately, the performance of the MSNS-AT score was compared with the SNS scoring system for the entire group and subgroups based on gestational age.

The mean MSNS-AT score (SD) is 13.6 (3.3) in preterm infants, 16.7 (2.4) in term infants, and 15.0 (3.3) for the entire study group. A significantly higher median MSNS-AT score (IQR) is found in preterm infants and in the entire group that survived as compared with those who died (14.0 (2.9) vs. 7.2 (2.0); *p* < 0.001 and 15.3 (3.0) vs. 9.5 (4.1); *p* < 0.001). The difference had limited significance in term infants (16.8 (2.4) vs. 14.8 (2.5); *p* = 0.050). Mann–Whitney U test showed a significant association of MSNS-AT with mortality only in preterm infants (median score 14, IQR 11–16; *p* = 0.042 in preterm infants; median 17 (IQR 15–18 in term infants, and 16 (13–17) in the whole group, *p* > 0.50 for both). However, an MSNS-AT score ≤ 10 demonstrates a statistically significant association with risk of death in the subgroups and the entire group when survivors are compared to non-survivors: an MSNS-AT score ≤ 10 is found in 21 survivors (10.3%) vs. 14 non-survivors (100%) in the preterm infants (*p* < 0.001); in 3 survivors (1.7%) vs. 1 non-survivor (16.7%) in the term infants group (*p* = 0.003; OR 9.1 [95%CI 1.25–61.13]), and in 24 survivors (6.3%) and 15 non-survivors (75%) in all patients (*p* < 0.001, OR 28.0 [10.7–72.89]).

**Figure 4:**
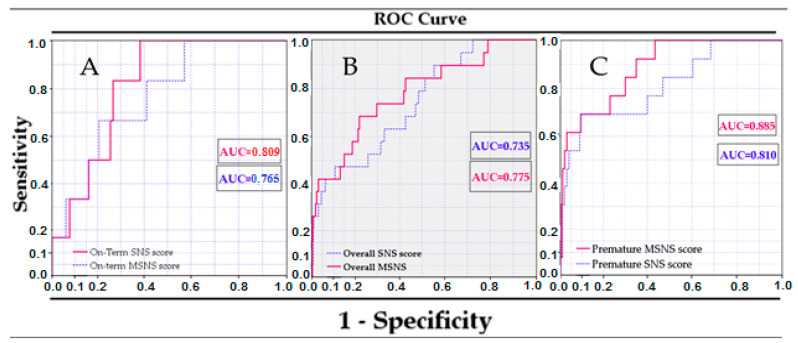
Differences between the SNS score and the novel MSNS-AT score in our study groups: (**A**) term newborns; (**B**) the entire group; (**C**) preterm infants.

The performance of the two scoring systems—SNS and MSNS-AT—was assessed by comparing the hazard probabilities of each multivariable model containing the scoring variable as a component and additional variables—gestational age, birth weight, time from birth to admission, Apgar score < 3 at 1 min, and early onset sepsis. Hazard probabilities were compared with ROC, and accuracy was determined by AUC for each model (Table 7). We found that our modified scoring system is more precise in predicting mortality compared with SNS (AUC 0.735 vs. 0.775) when applied to the entire group, irrespective of gestational age, and has lower accuracy in the term infants (AUC 0.765 vs. 0.809). The best accuracy for the prediction of all-cause mortality was found for both scoring systems in the preterm infants group, but again, the MSNS-AT score performed better than the SNS score (AUC 0.885 vs. 0.810) (Figure 4A–C).

### 3.4. Evaluation of Mortality Risk with MSNS-AT Score

**Figure 5:**
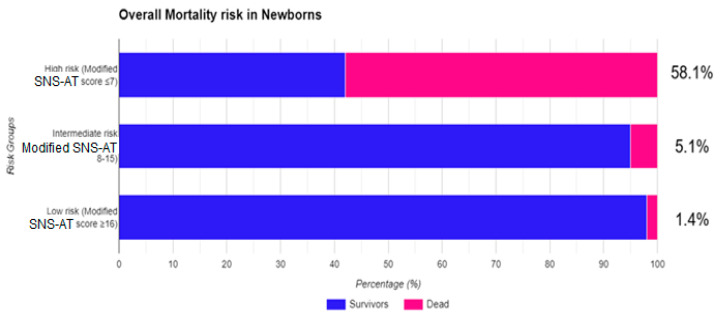
The overall risk of mortality assessed with MSNS-AT score.

To evaluate the risk of death using the MSN-AT score, we classified our patients based on the value of their MSNS-AT score. The first group has MSNS-AT scores ≤ 7, the second one comprises patients with scores between 8 and 15, and the last group has MSNS-AT scores ≥ 16 points. The Kaplan–Meyer curve was used to highlight differences in survival rates between groups. Significant survival disparities are found between the three groups classified according to MSNS-AT score (log-rank test: χ^2^ (2) =58.390; *p* < 0.001). Each group had a different risk of death, with the third group (MSNS-AT ≥ 16) having the smallest risk of mortality (Figure 5), as shown by multivariable Cox regression. Cox proportional hazards model shows that compared with the third group, the second one (MSNS-AT 8–15) has higher mortality (HR: 3.607; CI 95%: 1.023–14.653; *p* = 0.048) while the first group (MSNS-AT ≤ 7) has the highest mortality rate (HR: 47.120; CI 95%: 9.593–231.459; *p* < 0.001); adjustments for birth weight, gestational age, time to admission, Apgar score at 1 min, Apgar score under 3, and early onset sepsis were made in multivariable analysis (Table 8).

## 4. Discussion

One of the objectives of the World Health Organization’s (WHO) Millennium Development Goal (MDG) is to reduce under-five mortality by two-thirds. This includes a significant reduction in neonatal mortality, as it accounts for a large portion of under-five mortality. Despite a reduction of almost 50% of the global child mortality rates between 2000 and 2019, neonatal mortality rates did not decline as much, according to a recent report. In Romania, neonatal deaths declined from 884 (815–953) in 2015 to 690 (556–842) in 2019, the neonatal mortality rate reported in 2019 being 3.98, while the goal for 2030 is set at 3.17 [2]. Neonatal mortality is a serious concern as it reflects the health and economic status of a nation [3].

Regionalization of maternal and neonatal care and in-utero transportation of high-risk pregnancies to neonatal units with adequate resources and experience is the safest option for the best outcome both for mothers and newborns [6,7,8,9,10,11]. However, preterm births and delivery of sick infants are not always predictable, and these situations will continue to occur worldwide at lower-level institutions and even at home. It is estimated that in 50% of high-risk pregnancies, in-utero transportation is practically impossible [1]. On the other hand, 40% of neonatal deaths occur on the first day of life [30] and 75% in the first 7 days of life [27,34]. Delayed neonatal transfer, inadequate stabilization before transport, and deficient care during transportation are recognized as important risk factors for neonatal mortality and morbidity [5,6,35]. Experts consider neonatal transport between medical institutions as part of neonatal intensive care and a major outcome predictor for sick neonates [36]. Early recognition of sick neonates, optimal resuscitation if needed, prompt recognition and treatment of hypoglycemia, seizures, and respiratory distress, prevention of hypothermia, hypoxia, hypotension, adequate monitoring before and during neonatal transport, and rapid transfer are often challenging but extremely important for optimization of neonatal outcomes [5,8,9,13,14,15,16,30,35,37]. Efforts to improve neonatal stabilization pretransport, neonatal care during transport, and the neonatal transport system itself—organization, training, equipment—are challenging in many countries but mandatory for decreasing neonatal morbidity and mortality rates as data in the literature shows that outborn neonates are facing significantly higher morbidity and mortality rates [8,11,12]. Additionally, the regionalized, specialized neonatal transport system can reduce neonatal complications and improve survival rates [11,14,17,18]. However, neonatal transport of sick neonates has been and continues to be often neglected in low- and middle-income countries [7], as it is in Romania.

Regionalization of maternal and neonatal care started in Romania in 2002 with a first normative act establishing the criteria differentiating between level I, II, and III (the highest) maternity hospitals [31,38]. In 2004, the Romanian Ministry of Health issued another order for the experimental organization of three specialized neonatal transportation units [38,39]. Criteria for the regionalization of maternal and neonatal care were changed through normative governmental acts in 2009 and 2011, and another change is pending final approval by the end of this year [40]. For the future, five levels of maternity hospitals have been proposed. Neonatal transport has been neglected, so a new norm has been implemented, stating that each level III unit may have a special neonatal ambulance. This comes with special financial support from the National Recovery and Resilience Plan. In 2011, the Romanian Association of Neonatology developed a national guideline for pre-transport stabilization and transport of newborns [38]. According to current regulations, level I neonatal units must transfer all infants with a birth weight less than 2500 g, even healthy ones and all sick infants, to higher levels. Level II units can care for infants with a birth weight greater than 1500 g and/or a gestational age greater than 32 weeks, as long as they do not require mechanical ventilation or other invasive procedures. All other newborns must be transferred to level III units [31,32]. Our study group included 403 neonates after the exclusion of 15 infants submitted for severe congenital abnormalities, a rather large group of patients compared with most of the studies searching or evaluating a scoring system for the severity of the disease [20,26,27,28,29,30,41,42,43]. Out of the 403 infants in the study group, 217 were preterm infants (53.8%); 20 of the infants died, the fatality rate being 4.96% (Figure 1). Our maternity unit, part of an emergency county hospital, has been a level III regional maternal-neonatal unit since 2002, receiving between 70–90 outborn infants/year from one level II maternity hospital, 6 level I units, and after-delivery at home. The furthest inferior level I center is situated at 161 km, a trip of around 2 and a half hours by car. As shown in Figure 2, a significant drop in the number of submissions occurred after 2017, mostly due to an increased number of in-utero transfers. Increased awareness of high-risk pregnancies and their potential effects on maternal and neonatal outcomes leading to increased addressability of pregnant women directly to our center is another possible explanation. COVID-19 restrictions may have been added in 2020 and 2021.

Many authors [22,30,43,44,45,46] evaluated scoring systems separately for term and preterm infants, as gestational age and birth weight are recognized factors with significant impact on neonatal mortality. We had done the same, performing all the analysis on the entire group and in two subgroups based on gestational age—preterm and term infants. Some similarities and differences were noted starting the analysis of the baseline characteristics (Table 2): boys were slightly overrepresented in all three groups (over 50%), the proportions of infants with Apgar score < 3 at 1 min were almost equal in the groups, and most of the infants from each category were transferred from level I units. A subsequent diagnosis of early-onset sepsis was more often seen in term infants as compared with preterm ones (40.3% vs. 17.1%), preterm infants were more rapidly transferred as compared with term infants (mean duration 17.3 ± 65.0 vs. 27.0 ± 32.4 h), and death occurred more often in preterm infants (6.5% vs. 3.2%) (Table 2). Based on the regionalization of maternal and neonatal care, it is common for preterm infants to be taken to higher-level neonatal units. Because many of these infants face challenges transitioning to life outside the womb, this outcome is not surprising. Additionally, the delayed appearance of sepsis symptoms in term infants may be another explanation (also for delayed referral of term infants), but we did not collect data on transfer reasons and why term infants were transferred later than preterm infants.

Starting the 1990s, clinicians have struggled to find an objective, rapid tool to evaluate the severity of the disease in newborns, especially in outborn infants, as the severity of the disease was described as an important factor for neonatal morbidity and mortality [19,20,21,22] and neonatal transfer after delivery was found as a risk factor for increased morbidity and mortality in outborn infants as compared with inborn infants [12,35,47,48,49,50,51,52]. Many severity system scorings were developed, some based on clinical knowledge and some based on strong statistical associations between different clinical and laboratory variables and outcomes. Several problems have been cited related to almost all the scoring systems existing at this moment concerning what is expected from an ideal severity score. A perfect severity score, or predictive model for the severity of a disease, should be easily defined and applicable soon after admission. It should require minimally invasive procedures, be reproducible, and have a solid ability to predict mortality and specific morbidities. Additionally, it should be able to distinguish between neonates with varying outcomes [21,22,25,26,27,28,37]. All such scoring systems need accurate validation in reasonably large data sets, calibration, tests for their discrimination capacity (scores with AUC >0.8 are useful in practice), reproducibility, and capacity to avoid biases [25]. Scores such as CRIB, CRIB II, SNAP, SNAP-PE, SNAP II, SNAPPE II, TISS and NTISS, NICHHD, NMPI, NBRS, TOPS, TRIPS, MINT, Prem or Berlin score, and Sinkin score are complex scores, with different power to predict mortality, either comprising multiple parameters, either designed for a specific population (e.g., preterm infants) or special situation [3,9,11,19,20,22,25,31,38,43,44,45,46,50,51,52,53,54,55]. We had chosen the sick neonate score (SNS) score, a score initially developed by Hermansen in 1994 [33] and modified by Rathod et al. [30] since this score was validated both in high-income countries and in resource-limited countries [30,33], and, after exclusion of pH and partial oxygen pressure from Hermansen score, no invasive procedure was required. Additionally, in many studies, an SNS score ≤ 8 has been validated as a cut-off value for predicting mortality [29,30,56]. We calculated the SNS score (Table 2) using rectal temperature instead of axillary temperature because it is more accurate. The mean values of SNS score were comparable between study groups (10.0 ± 2.6 in preterm infants, 11.8 ± 2.2 in term infants, and 10.8 ± 2.6 in the whole group). Significantly lower SNS scores were found when we compared the survivors and non-survivors in all study groups (comparison of median (IQR) values in Table 3 and Table 4; all *p* < 0.05), but the SNS cut-off value ≤ 8 was associated with death only in preterm infants and the entire study groups (Table 5). We believe that the nonsignificant association of this cut-off value in term infants is due to the low number of term infants who died (5).

The next step was finding new variables easy to use for the SNS score to improve its ability to predict mortality in all neonatal populations. Lower gestational age and birth weight were repeatedly demonstrated as associated with an increased risk of death; therefore, we used them for the development of a new scoring system, the same as other authors [5,26,27,28,57]. Gender, early onset sepsis diagnosis, and asphyxia (defined as an Apgar score < 3/1 min) showed no association with mortality (*p* > 0.05) in our statistics (Table 5). A clinical observation during the years was that infants submitted rapidly after delivery have a better course; therefore, we tried to statistically analyze the prediction power of time to admission upon the mortality rate, and the statistical analysis confirmed that time to admission was significantly associated with mortality in preterm infants and in the whole group (Table 4) and a duration of 6.5 h between birth and admission into our unit had an AUC of 0.664, *p* = 0.013, the sensitivity of 85% and specificity of 54% in predicting mortality (Figure 3). Mori et al. found that neonatal transport duration over 90 min is associated with a two times higher risk of death [58]. A new scoring model was designed (Table 6) using the SNS score and new variables, gestational age, birth weight, and time to admission, and we named it MSNS-AT. Evaluation of MSNS-AT scores in our study groups was encouraging. MSNS-AT score ≤ 10 demonstrated a statistically significant association with risk of death in the subgroups and in the entire group, was found in 21 survivors (10.3%) vs. 14 non-survivors (100%) in the preterm infants (*p* < 0.001); in 3 survivors (1.7%) vs. 1 non-survivor (16.7%) in the term infants group (*p* = 0.003; OR 9.1 [95%CI 1.25–61.13]), and in 24 survivors (6.3%) and 15 non-survivors (75%) in all patients (*p* < 0.001, OR 28.0 [95% CI 10.7–72.89]). These results are consistent with those reported in Table 4 (comparison of the median AT and corresponding IQR) in the study group. Larger IQR intervals were seen in term infants, and this most probably influenced the IQR range for the entire group. As expected, preterm infants were transferred sooner than term ones, reflecting good compliance with the national recommendations, but this significantly influenced the CI when a comparison was performed. The results are comparable with those using MSNS to predict mortality. Padar et al. [26] found that a cut-off value of 10 predicted mortality with a sensitivity of 88.24% and sensibility of 92.5% in 248 neonates evaluated at birth and at 24 h of life. A mean score of 9.11 was found in non-survivors as compared with 12.9 survivors in a group of 71 newborns, of which 80% were term infants in a study published by Reddy and colleagues [27]. A bigger study performed by Mansoor et al. [57], including 585 neonates, both inborn and outborn infants, found a mean score of 8.2 ± 2.96 in deceased newborns vs. 13.1 ± 2.4 in survivors, while the cut-off value of 10 had 90% sensitivity and 88% specificity for predicting mortality. The same cut-off value predicted death with 85.9% sensitivity and 51.1% specificity in another recent study of 355 neonates but identified that better prediction of mortality can be done using a cut-off value of 8 [28]. Adding time to admission to our score, we expected that the mean MSNS-AT would be higher compared with MSNS, but we still found significant differences in the score between survivors and non-survivors in preterm infants and for the entire group: 14.0 ± 2.9 vs. 7.2 ± 2.0 (*p* < 0.001) and 15.3 ± 3.0 vs. 9.5 ± 4.1 (*p* < 0.001), respectively. Limited significance was found in term infants (16.8 ± 2.4 vs. 14.8 ± 2.5; *p* = 0.050), probably because most term infants arrived significantly later at our unit. Obviously, time to admission may be influenced by multiple factors, many of them on the administrative level. Lack of specialists at lower level neonatal units, able to timely recognize sick neonates in need of specialized care, limited availability of transport teams and ambulances, and insufficient experience in carrying sick neonates during transportation are just some of the factors that may influence time to admission in outborn infants and, subsequently, their outcomes.

Finally, the MSNS-AT score was tested for accuracy in all the study groups, adjusting for gestational age, birth weight, Apgar score < 3, and early onset sepsis rate, and in comparison with the SNS score (Table 7, Figure 4 and Figure 5) and we found that this new score has the better performance in predicting mortality vs. SNS score in the whole group, irrespective of gestational age (AUC 0.735 vs. 0.775) and performed even better in preterm infants (AUC 0.885 vs. 0.810). A lower accuracy was found in term infants (MSNS-AT AUC 0.765 vs. SNS AUC 0.809). Another analysis has shown that a cut-off value ≤ 7 accurately predicted death in 58.1% of the infants in the study group, while only 1.4% of infants with values > 16 have died. Due to the low number of deaths (the main outcome of our study) both in preterm and in term infants groups, we expected a lack of linearity in the ROC curves. However, the results were not influenced since, overall, only a few infants died. The overall mortality rate of the group (4.96%) is comparable with the rate cited by other studies [3,12,48,54].

Severity scores were developed accordingly, including clinical and laboratory parameters, with different utilities and results in predicting mortality and morbidity rates [22]. Additionally, not all these scores can be applied in resource-limited areas. Still, an ideal score was not identified, and this may be difficult even now because validation of various scores has not demonstrated the same sensibility and specificity in different neonatal populations, from different regions, countries, and neonatal units, with so many organizational differences between national maternal and neonatal care regionalization and neonatal transport systems [5,23,25]. Currently, no unique mathematical formula can completely capture the complex clinical neonatal process, regardless of the accuracy of the scoring system, according to some experts [5].

However, these predictions allow better planning and usage of resources of care, improvements of neonatal care before and during transport, cost analysis, evaluation of the care quality, comparisons between neonatal units, research, and even parental counseling [8,22,25,26,27,28,29,53]. Therefore, clinicians like us will continue searching for the ideal scoring system, ideal at least for the population they have to care for. We made efforts to find the best—in terms of accuracy—and easiest-to-apply scoring system for our population, based on our experience that preterm infants have to be considered and analyzed separately from term infants.

We acknowledge some limitations of our study. We did not evaluate the individual components of the SNS score as this was not the goal of our study. Instead, we plan to do so in a future study in which we plan to compare SNS scores before transfer and at arrival in our unit to evaluate pretransport stabilization and the quality of care during neonatal transport. Additionally, probably a higher number of patients would give increased statistical power to some of our comparisons and associations. The study period was quite long—7 years—and protocols for stabilization and transport have been changed during this period, influencing the neonatal status at arrival in some of the newborns included in the study. Another limitation of our study, when comparing our results to the results of other studies, might be the fact that we used a slightly modified SNS score for the initial evaluation of our study groups. However, using the first blood glucose instead of a random blood glucose value may increase the predictive value of the SNS score as it may better reflect the condition of the newborns at admission at our hospital and exclude corrective interventions (including parenteral or enteral administration of glucose). Choosing the first blood glucose value may help diagnose hypoglycemia, a significant risk factor for neonatal morbidity and mortality [5,9,13,30]. Using Masimo technology for peripheral oxygen saturation measuring aimed at a rapid and accurate evaluation of the infants’ condition and identification of hypoxia, another important risk factor for morbidity and mortality [5,9,13,30]. We did not perform the yearly comparative analysis of the severity scores. The accuracy of our new MSNS-AT score for mortality prediction was verified in the study group, but a validation of this new score and its predictive accuracy in a new cohort is mandatory and planned by the authors.

## 5. Conclusions

The MSNS-AT score—calculated by adding gestational age, birth weight, and time to admission to the SNS score—significantly improved mortality prediction at admission in the whole study group as compared with the SNS score. We found, as other authors, that the predictive accuracy of the same score is different in term and preterm infants, suggesting that different scores have to be used for these categories of neonates. The best accuracy prediction of the MSNS-AT score was observed in the group of preterm infants, suggesting that, besides gestational age and birth weight, time to admission may be decisive for the outcome of outborn preterm infants. Further studies are necessary to confirm the predictive value of the MSNS-AT score and to identify variables that can improve its value in term infants without affecting the simplicity, ease, and rapidity of the scoring system. We are planning to validate the new score in a new, independent data set.

Based on a prospective cohort starting from the SNS score and using statistical models, we developed an MSNS-AT score with better predictive performance for mortality in outborn infants that may help us to better allocate all types of resources and to adjust care and treatment to optimize neonatal outcomes. The organization of the Romanian neonatal transport system started in 2004, immediately after the regionalization of maternal and neonatal care; at that time qualified, specialized staff and special equipment were limited or even lacking in many parts of the country. Considering the improved rates of survival at lower gestational ages and the insufficient number of beds in neonatal intensive care units (NICU), we face a continuous need for better critical neonatal care, as survival is not the ultimate goal of NICU care anymore.

## Figures and Tables

**Table 1:** Sick neonatal score [30].

Variables	Score		
	0	1	2
Respiratory effort	Apnea or grunting	Tachypnea (>60/min) +/− retractions	Normal (40–60/min)
Heart rate	Bradycardia/ Asystole	Tachycardia (>160/min)	Normal (100–160/min)
Mean blood pressure (mmHg)	<30	30–39	>39
Axillary temperature (°C)	<36	36–36.5	36.5–37.5
Capillary filling time (s)	>5	3–5	<3
Random blood sugar (mg/dL)	<40	40–60	>60
SpO_2_ in room air (%)	<85	85–92	>92

**Table 2:** Baseline characteristics of the study group and subgroups.

	Preterm Infants (N = 217)	Term Infants (N = 186)	All Infants (N = 403)
Gestational age (weeks) (mean/SD/median + IQR)	35.56 ± 2.87/33 (31–35)	38.73 ± 1.31/39 (38–39)	35.40 ± 3.83/36 (33–39)
Birth weight (grams) (mean/SD/median + IQR)	1788.55 ± 561.90/ 1750 (1400–2162.5)	3119.30 ± 631.25/ 3100 (2800–3422.5)	2402.74 ± 891.80/ 2330 (1700–3100)
Male gender (N/%)	113/52.1	111/59.7	224/55.6
Time to admission (hours) (mean/SD/median + IQR)	17.33 ± 65.07/4 (3–7)	27.06 ± 32.44/12.5 (5–36)	21.82 ± 52.75/6 (3–21)
Apgar score/1 min (mean/SD/median + IQR)	6.60 ± 2.12/7 (6–8)	7.30 ± 2.32/8 (6.75–9)	6.92 ± 2.24/7 (6–8.75)
Apgar score < 3/1 min (N/%)	22/10.1	19/10.2	41/10.2
Early onset sepsis (N/%)	37/17.1	75/40.3	112/27.8
Place of birth			
Home (N/%)	8/3.7	5/2.7	13/3.2
Level I (N/%)	125/57.6	126/67.7	251/62.3
Level II (N/%)	83/38.2	55/29.6	138/34.3
Level III (N/%)	1/0.5	-	1/0.2
Death (N/%)	14/6.5	6/3.2	20/5.0
SNS score (mean/SD/median + IQR)	10.04 ± 2.67/11 (8–12)	11.85 ± 2.20/12.5 (10–16)	10.87 ± 2.63/11 (9–13)

**Table 3:** Comparison of baseline characteristics between survivors and non-survivors in the whole group and subgroups based on gestational age.

	Preterm Infants (N = 217)		Term Infants (N = 186)		All Infants (N = 403)	
	Survivors (Mean/SD)	Non-Survivors (Mean/SD)	Survivors (Mean/SD)	Non-Survivors (Mean/SD)	Survivors (Mean/SD)	Non-Survivors (Mean/SD)
Gestational age (weeks)	32.7/2.7	30.7/4.4	38.7/1.3	38.3/1.2	35.5/3.7	32.9/5.1
Birth weight (grams)	1804/543	1557/767	3128/638	2853/261	2426/885	1946/889
Time to admission (hours)	16.7/66.3	26.1/44.2	26.8/32.6	34.7/29.0	21.5/53.4	28.7/39.7
Apgar score/1 min	6.6/2.2	5.7/2.0	7.3/2.4	8.3/1.6	6.9/2.3	6.5/2.2
SNS score	10.4/2.4	5.3/2.1	11.9/2.2	10.2/2.2	11.1/2.4	6.8/3.0

**Table 4:** Association of tested variables with mortality in study groups.

	Preterm Infants (N = 217)		Term Infants (N = 186)		All Infants (N = 403)	
	Median (IQR)	*p* *	Median (IQR)	*p* *	Median (IQR)	*p* *
Gestational age (weeks)	36 (31–35)	0.077	39 (38–39)	0.525	36 (33–39)	0.026
Birth weight (grams)	1750 (1400–2162.5)	0.164	3100 (2800–3422.5)	0.135	2330 (1700–3100)	0.032
Time to admission (hours)	4 (3–7)	<0.001	12.5 (5–36)	0.343	6.0 (3–21)	0.013
Apgar score/1 min.	7 (6–8)	<0.001	8 (6.75–9)	0.038	7 (6–8.75)	<0.001
SNS score	11.0 (8–12)	<0.001	12.5 (10–14)	0.038	11.0 (11–13)	<0.001

* Mann–Whitney U test.

**Table 5:** Baseline characteristics and their association with mortality.

	Preterm Infants				Term Infants				All			
	Survivors (N/%)	Non-Survivors (N/%)	*p*-Value	OR (95%CI)	Survivors (N/%)	Non-Survivors (N/%)	*p*-Value	OR (95%CI)	Survivors (N/%)	Non-Survivors (N/%)	*p*-Value	OR (95%CI)
Male gender	105/51.7	8/57.1	0.696	1.23 (0.44–3.42)	106/58.9	5/83.3	0.232	3.38 (0.46–28.34)	211/55.1	13/65.0	0.386	1.48 (0.60–3.64)
Apgar score < 3	20/9.9	2/15.4	0.529	1.65 (0.34–7.99)	19/10.6	0/0	0.404	-	39/10.2	2/10.5	0.995	1.03 (0.23–4.65)
Early sepsis	34/16.7	3/21.4	0.654	1.36 (0.36–5.12)	72/40.0	3/50.0	0.626	1.50 (0.29–7.64)	106/27.7	6/30.0	0.822	1.12 (0.42–2.99)
SNS score ≤ 8	48/23.6	14/100	<0.001	1.29 (1.13–1.48)	18/10.0	1/16.7	0.598	1.80 (0.20–16.27)	66/17.2	15/75.0	<0.001	14.41 (5.06–41.02)
Place of birth												
Home	7/3.4	1/7.1	0.013	-	5/2.8	0/0	0.750	-	12/3.1	1/5.0	0.014	-
Level I	123/60.6	2/14.3			122/67.8	4/66.7			245/64.0	6/30.0		
Level II	72/35.5	11/78.6			53/29.4	2/33.3			125/32.6	13/65.0		
Level III	1/0.5	0/0			0/0	0/0			1/0.3	0/0		

**Table 6:** Modified SNS-Admission Time (MSNS-AT) score.

	0 Points	1 Points	2 Points	3 Points
Gestational age (weeks)	<32	32–36	≥37	-
Birth weight (g)	<1500	1500–2499	≥2500	-
Time from birth to admission (h)	≥12	6–12	-	<6
Final MSNS-AT score	Points granted on the above variables are added to the SNS score			

**Table 7:** Accuracy of the two scoring systems for prediction mortality evaluated in study groups.

Model	AUC	*p*-Value	95% CI	
			Lower	Upper
SNS score in term infants	0.809	0.010	0.698	0.920
MSNS-AT in term infants	0.765	0.027	0.601	0.929
SNS score in preterm infants	0.810	0.000	0.676	0.945
MSNS-AT in preterm infants	0.885	0.000	0.800	0.970
SNS score in all infants	0.735	0.001	0.622	0.848
MSNS-AT in all infants	0.775	0.000	0.659	0.890

**Table 8:** Cox proportional multivariable model with adjustments.

Multivariable Cox Regression	*p*-Value	HR	95.0% CI for HR	
Variables			Lower	Upper
Gestational age	0.920	1.012	0.800	1.280
Birth weight	0.904	1.000	0.999	1.001
Apgar 1 min	0.563	1.113	0.774	1.600
Apgar < 3	0.966	1.054	0.093	11.918
Early sepsis	0.871	0.917	0.322	2.610
Time-to-admission	0.575	1.001	0.997	1.006
MSNS-AT ≥ 16	<0.001			
MSNS-AT 8–15	0.048	3.607	1.023	14.653
MSNS-AT ≤ 7	<0.001	47.120	9.593	231.459

## Data Availability

The data presented in this study are not publicly available due to institutional restrictions but are available upon reasonable request from the corresponding author.
